# Polygenic risk for ADHD and ASD and their relation with cognitive measures in school children

**DOI:** 10.1017/S0033291720003189

**Published:** 2022-05

**Authors:** Sofía Aguilar-Lacasaña, Natàlia Vilor-Tejedor, Philip R. Jansen, Mònica López-Vicente, Mariona Bustamante, Miguel Burgaleta, Jordi Sunyer, Silvia Alemany

**Affiliations:** 1 University of Vic – Central University of Catalonia (UVIC-UCC), Vic, Spain; 2 Barcelona Research Institute for Global Health (ISGlobal), Barcelona, Spain; 3 Centre for Genomic Regulation (CRG), The Barcelona Institute of Science and Technology, Barcelona, Spain; 4 Barcelona Beta Brain Research Center (BBBRC), Pasqual Maragall Foundation, Barcelona, Spain; 5 Department of Clinical Genetics, ERASMUS MC, Rotterdam, The Netherlands; 6 Department of Complex Trait Genetics, Center for Neurogenomics and Cognitive Research, Amsterdam Neuroscience, VU University and Department of Clinical Genetics, Amsterdam UMC - location VUmc, Amsterdam, The Netherlands; 7 Department of Child and Adolescent Psychiatry/Psychology, Erasmus University Medical Center, Rotterdam, The Netherlands; 8 Universitat Pompeu Fabra (UPF), Barcelona, Spain; 9 CIBER Epidemiología y Salud Pública (CIBERESP), Spain; 10 Department of Technology, Center for Brain and Cognition, Universitat Pompeu Fabra, Roc Boronat 138, 08018 Barcelona, Spain; 11 IMIM (Hospital del Mar Medical Research Institute), Barcelona, Spain

**Keywords:** ADHD, ASD, cognition, polygenic risk score, working memory

## Abstract

**Background:**

Attention deficit and hyperactivity disorder (ADHD) and autism spectrum disorder (ASD) are child-onset neurodevelopmental disorders frequently accompanied by cognitive difficulties. In the current study, we aim to examine the genetic overlap between ADHD and ASD and cognitive measures of working memory (WM) and attention performance among schoolchildren using a polygenic risk approach.

**Methods:**

A total of 1667 children from a population-based cohort aged 7–11 years with data available on genetics and cognition were included in the analyses. Polygenic risk scores (PRS) were calculated for ADHD and ASD using results from the largest GWAS to date (*N* = 55 374 and *N* = 46 351, respectively). The cognitive outcomes included verbal and numerical WM and the standard error of hit reaction time (HRTSE) as a measure of attention performance. These outcomes were repeatedly assessed over 1-year period using computerized version of the Attention Network Test and *n*-back task. Associations were estimated using linear mixed-effects models.

**Results:**

Higher polygenic risk for ADHD was associated with lower WM performance at baseline time but not over time. These findings remained significant after adjusting by multiple testing and excluding individuals with an ADHD diagnosis but were limited to boys. PRS for ASD was only nominally associated with an increased improvement on verbal WM over time, although this association did not survive multiple testing correction. No associations were observed for HRTSE.

**Conclusions:**

Common genetic variants related to ADHD may contribute to worse WM performance among schoolchildren from the general population but not to the subsequent cognitive-developmental trajectory assessed over 1-year period.

## Introduction

Attention-deficit/hyperactivity disorder (ADHD) and autism spectrum disorder (ASD) are complex neurodevelopmental disorders that emerge during childhood (Lord, Elsabbagh, Baird, & Veenstra-Vanderweele, 2018; Thapar & Cooper, 2016). Several aspects are common to both conditions including early onset, delays and alterations in brain development, a male preponderance and cognitive difficulties (Visser, Rommelse, Greven, & Buitelaar, 2016).

In this regard, cognitive performance of individuals with ADHD has been found to be consistently lower compared to controls for several domains such as working memory (WM) or variability in reaction time (RT), which reflects attentional lapses (Karalunas, Geurts, Konrad, Bender, & Nigg, 2014; Pievsky & McGrath, 2018). Remarkably, cognitive performance has been related to later symptom severity and functional outcomes among ADHD patients in a longitudinal study (van Lieshout et al., 2017). Furthermore, cognitive deficits are related to ADHD symptoms beyond clinical status. In a population-based sample study, inattention symptoms were related to deficits in WM (Tillman, Eninger, Forssman, & Bohlin, 2011). In the case of ASD, although some individuals exhibit cognitive strengths in certain domains (Iuculano et al., 2014), impairments have been reported in functions such as WM or attention (Boxhoorn et al., 2018; Karalunas et al., 2018; Velikonja, Fett, & Velthorst, 2019; Willcutt, Sonuga-Barke, Nigg, & Sergeant, 2008).

Family and twin studies indicate that common familial/genetic factors are underlying these cognitive functions, ADHD and possibly ASD (Cheung, Fazier-Wood, Asherson, Rijsdijk, & Kuntsi, 2014; Frazier-Wood et al., 2012; Michelini et al., 2018). In line with these findings, it has been suggested that cognitive difficulties, social-communication behavioral traits and ADHD symptoms are underpinned at a biological level, which may involve genetic variants related to these disorders (Martin, Hamshere, Stergiakouli, O'Donovan, & Thapar, 2015). This can be examined by computing polygenic risk scores which index genetic susceptibility for a given disorder (Wray et al., 2014). Polygenic risk scores can then be used to assess shared genetic influences across different traits (Choi et al., 2020).

So far, a few studies have used polygenic risk scores to investigate the potential genetic overlap between ADHD and ASD and cognitive deficits (Clarke et al., 2016; Du Rietz et al., 2018; Schork et al., 2019; Stergiakouli et al., 2017). A study investigating polygenic risk for ASD and ADHD in a large population-based sample found that ASD-related variants may confer cognitive advantages, while the polygenic risk for ADHD did not show a consistent pattern of results for the cognitive domains analyzed (Clarke et al., 2016). Another study also found evidence suggesting that polygenic risk for autism is related with better performance in executive function (*N* = 417) but these findings were not replicated in an independent sample (*N* = 3681) (Schork et al., 2019). Other studies in children, adolescents and adults showed negative associations between polygenic risk for ADHD and cognitive and school performance (Du Rietz et al., 2018; Martin et al., 2015; Stergiakouli et al., 2017). Thus, it is still not clear whether the manifestation of polygenic risk for these neurodevelopmental conditions involves cognitive deficits in population-based samples. Furthermore, none of these studies investigated attention performance, a core aspect of ADHD (Barkley, 1997).

In the current study, we examined whether polygenic risk for ADHD and ASD was associated with baseline and 1-year cognitive-developmental trajectories of measures of WM and attention performance among school-aged children from the general population.

## Material and methods

### Study population

Participants were drawn from the BREATHE project (European Commission: FP7-ERC-2010-AdG, ID 268479), a population-based cohort of primary school-aged children designed to analyze associations between air pollution and behavior, cognitive abilities and brain structure and function. A full description of the project is available elsewhere (Sunyer et al., 2015). The project was conducted in 39 schools in or near Barcelona (Spain). All families of children without special needs who were enrolled in second, third and fourth grades at the selected schools were invited to participate in the study. A total of 2897 children participated in the project for which genotype data was available in 1667 children of European ancestry.

All parents or legal guardians gave written informed consent, and the study was approved by the IMIM-Parc de Salut Mar Research Ethics Committee (No. 2010/41,221/I), Barcelona, Spain; and the FP7-ERC-2010-AdG Ethics Review Committee (268,479–22,022,011).

### Cognitive measures

The outcomes of the study were WM and attention performance. WM was assessed using the *n*-back task (Nelson et al., 2000). A full description of the task can be found in Methods S2. Briefly, in the *n*-back task, a series of stimuli are presented individually at the center of the screen. Participants were required to monitor those items and indicate whenever the stimulus matched the one presented 1, 2 or 3 stimuli back, also known as back loads. Here, we used numbers and words as stimuli in the 3-back level and we calculated *d* prime (*d′*) as an indicator of WM accuracy. These measures, 3-back numbers *d′* and 3-back words *d′* were treated in the analysis as indicators of verbal and numerical WM performance when using words and numbers, respectively, as stimuli. These measures were analyzed as continuous variables. Higher *d*′ scores indicated more accuracy and thus, better performance.

Attention performance was assessed using the computerized version of the Attention Network Test [ANT, (Rueda et al., 2004)]. A full description of the test can be found in Methods S1. Briefly, reaction time measures (i.e. the time between the introduction of a stimulus and the reaction on the subject to that stimulus) were used to calculate the different outcomes that can be obtained with the ANT. The outcome analyzed herein corresponds to the hit reaction time standard error (HRTSE) (standard error of reaction time for correct responses), a measure of intraindividual variability reflecting response speed and consistency throughout the test. HRTSE was analyzed as a continuous variable with lower scores indicating better performance.

The participants completed these tests repeatedly through four different sessions (every 3 months) over 1 year. We used these four repeated measures to model the 1-year developmental trajectories of verbal and numerical WM and HRTSE. The modeled 1-year trajectory may include 1–4 repeated measures of the cognitive measures based on available data.

### Behavioral measures

To validate the PRS in our cohort, behavioral data were used to test the association between the PRS and the phenotype. Behavioral measures were obtained at the beginning of the cognitive data collection (visit 1, baseline). ADHD symptoms were assessed using a questionnaire based on the ADHD diagnostic criteria described in Diagnostic and Statistical Manual of Mental Disorders 4th edition [DSM-IV (American Psychiatric Association., 2002)], and was completed by teachers. The ADHD-DSM-IV questionnaire consists of a list of 18 symptoms, assessing two separate symptom groups: inattention (nine symptoms) and hyperactivity/impulsivity (nine symptoms). Each ADHD symptom is rated on a 4-point frequency scale from never or rarely (0) to very often (3). The ADHD symptom score ranges from 0 to 54, with higher scores indicating more symptoms. ASD symptoms were not collected in this project.

### Genotyping

DNA samples were obtained from saliva and genome-wide genotyping was performed using the HumanCore BeadChipWG-330-1,101 (Illumina). A full description of the genotyping and quality control procedures is available elsewhere (Alemany et al., 2016) and summarized in Methods S3.

### Polygenic scoring

Genotyped data that passed quality control were used to compute PRS using PRSice (Euesden, Lewis, & O'Reilly, 2015). PRSice calculates individual polygenic scores by summing up all the SNP alleles carried by the participants weighted by the SNP allele effect size estimated in a previous GWAS. PRS based on five different *p* value thresholds (*P_T_*) for SNP inclusion *P_T_* *=* {0.01, 0.05, 0.1, 0.5, 1} were calculated for each disorder. Polygenic scoring was performed in clumped variants (representative genetic variants per linkage disequilibrium block) using *r*^2^ > 0.1 as a cut-off for LD independent markers in a 250-kb window. This means that SNPs with the smallest *p* value in each 250 kb window is retained and all those in LD (*r*^2^ > 0.1) with these SNPs are removed. All PRS were standardized to a mean of 0 and a standard deviation of 1. The number of variants included in the PRS for each *P_T_* can be found in online Supplementary Table S1.

PRS were based on the most recent ASD and ADHD GWAS. The ASD GWAS included 46 351 individuals (18 382 cases) of European ancestry and 9 112 387 SNPs (Grove et al., 2019). The ADHD GWAS included 53 293 individuals (19 099 cases) of European ancestry and 8 047 421 SNPs (Demontis et al., 2019).

### Statistical analysis

**Fig. 1.**
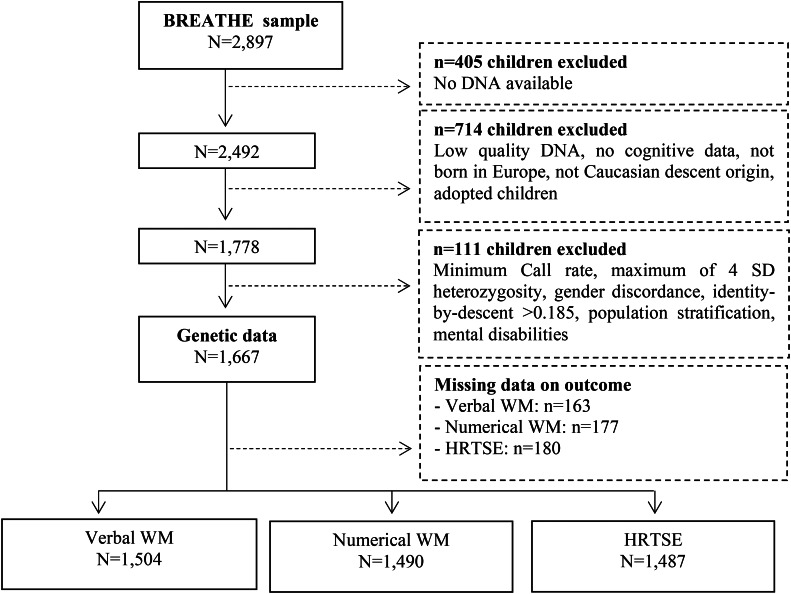
Flow chart depicting the final sample size for the outcomes analyzed including hit reaction time (HRT-SE) of ANT as a measure of HRTSE and *d′* values from the 3-back task with words and numbers as stimuli as measures of WM. Solid lines and boxes represent individuals remaining in the study, dashed lines and boxes represent individuals excluded. Reason and number of individuals excluded are indicated in dashed boxes.

The final data included 1667 children with complete data on PRS, verbal WM (*N* = 1504, representing 6130 observations), numerical WM (*N* = 1490, representing 6100 observations) and HRTSE (*N* = 1487, representing 6177 observations) (Fig. 1). Of these, 80% had data on at least three of the four repeated assessments. To validate the PRS, we tested the association between the PRS and the behavioral manifestation of ADHD. We estimated the associations between PRS for ADHD across all significance thresholds and the behavioral measure using linear mixed-effects models with the school as nested random effect. A total of 1555 individuals were included in these analyses.

We tested the cross-sectional association between polygenic risk for ADHD (PRS-ADHD) and ASD (PRS-ASD) and cognitive measures using linear mixed-effects models with the school as a nested random effect. These associations were tested using the cognitive outcomes assessed at baseline. The baseline measures correspond to those obtained in visit 1 when participants completed the cognitive tasks for the first time.

In the validation and cross-sectional analysis, we included schools as nested random effects to account for the multilevel nature of the data (i.e. children within schools).

We analyzed the association between PRS-ADHD and PRS-ASD and changes in cognitive measures repeatedly assessed over 1-year period (1-year trajectories) using linear mixed-effects models with individual children nested within schools as random effects. An interaction between age (centered at visit 1) at each visit and the PRS was included to capture changes in 1-year trajectory associated with polygenic risk for ADHD or ASD as shown below.
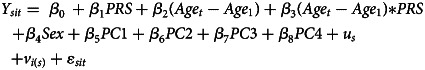
where *Y_sit_* is the test score of the cognitive measure for the individual *i* in school *s* at visit *t*, *t* = {1,2,3,4}; *u_s_* is random effects at the school level, *v_i_*_(*s*)_ is random effects associated with individual *i* in school *s* and *ε_sit_* is the model residuals. Only participants with data on at least two visits (representing around 85% of the sample) were included in the analyses of 1-year trajectories. Thus, the modeled 1-year trajectory may include two–four repeated measures of the cognitive outcomes per individual. Of note, around 73% of the participants have data for the four visits. Previous research in the BREATHE entire sample (*n* = 2897) have shown age-related growth patterns on both WM and ANT measures over the 1-year period (López-Vicente et al., 2016; Suades-González et al., 2017). These findings suggest that the repeated measures through the year were capturing developmental trajectories where the accuracy of the tasks improved with age. These trajectories are likely to reflect cognitive maturation processes rather than practice effects because no important differences were observed in the median scores of children at same age assessed in different sessions (López-Vicente et al., 2016; Mollica, Maruff, Collie, & Vance, 2005).

Because ADHD and ASD present higher prevalence among boys (Lord et al., 2018; Polanczyk, De Lima, Horta, Biederman, & Rohde, 2007), we explored PRS interaction by sex by fitting an interaction term in each model. Stratified models by sex are presented when significant interactions between PRS and sex were detected.

In addition, we performed sensitivity analyses, in which we excluded individuals with ADHD diagnosis made by a medical doctor. Clinical status of other psychiatric disorders was not collected in the study.

In all models, PRS were the determinant and cognitive measures were the outcome. Cognitive measures included verbal and numerical WM and HRTSE. All models were adjusted by sex, age and the first four genetic principal components. One model for each *P_T_* was constructed.

Effect sizes were reported as beta coefficients throughout the results. Baseline and trajectory analysis were corrected for multiple testing across all PRS, generated at five different significance thresholds, tested for association with three cognitive outcomes using the false discovery rate (FDR) method (Benjamini & Hochberg, 1995). Results at *p* uncorrected <0.05 were considered nominally significant and results at *p* FDR<0.05 were considered statistically significant.

All statistical analyses were conducted using the R statistical software (version 3.6.0).

## Results

### Descriptive results

Descriptive characteristics of the subjects included in the analyses are presented in Table 1. The mean age of the sample was 8.5 ± 0.9 years old, 46.9% were female and 61.4% of the mothers have higher education (Table 1). In comparison with the participants of the BREATHE project for whom genetic data were not available (*n* = 1230), children who were included were significantly more likely to be male and presented better verbal and numerical WM performance as well as less HRTSE. Regarding behavioral measures, children included had lower inattention and total ADHD symptoms. There were no significant differences in terms of age or hyperactivity symptoms (online Supplementary Table S2). Boys performed better for numerical WM and HRTSE measures and presented higher total ADHD, inattention and hyperactivity symptoms (online Supplementary Table S3).

**Table 1.** Characteristics of the sample

Characteristic	Study sample (*n* = 1667)
Sex (female), *n* (%)	782 (46.9)
Age (years), mean (s.d.)	8.5 (0.9)
Cognitive measures	
Verbal WM, mean (s.d.)^a^	1.4 (1.0)
Numerical WM, mean (s.d.)^a^	1.3 (1.0)
HRTSE, mean (s.d.)	267.2 (88.0)
Behavioral measures	
ADHD symptom scores, mean (s.d.)	7.7 (9.4)
Inattention, mean (s.d.)	4.6 (5.6)
Hyperactivity, mean (s.d.)	3.1 (4.9)

NOTE: ADHD total symptoms score, teacher-reported attention deficit hyperactivity disorder symptoms where higher scores indicate more symptoms. HRTSE, standard error of the hit reaction time obtained from the Attentional Network Test, higher scores indicate worse attention performance. WM, working memory performance (*d*’ values) from the 3-back task of the *n*-back test with words (Verbal) and numbers (Numerical) as stimuli, higher values indicate better WM.

a Baseline (visit 1).

Online Supplementary Fig. S1 shows the correlations between cognitive measures in each visit and online Supplementary Fig. S2 summarizes the correlations between the PRS for ADHD and ASD. Measures within each domain (i.e. WM and HRTSE) were positively correlated across the four visits. WM and HRTSE measures were negatively correlated, which indicates that better performance in the first domain (high *d′*) is related to better performance in the second (low HRT-SE).

### Validation using behavioral measures

Polygenic risk for ADHD was positively associated with ADHD symptoms total score and hyperactivity at all significance thresholds. Positive associations between polygenic risk for ADHD and inattention were observed at all significance thresholds but were not significant at *P_T_ <*0.01 (online Supplementary Table S4).

### Cross-sectional association between PRS and cognitive performance

PRS for ADHD were negatively associated with verbal WM across all significance thresholds with the strongest association observed at *P_T_* = 1 (*β* = −0.086, 95% CI = −0.139, −0.033; *p-uncorrec.* = 0.002, *p*-FDR = 0.026) (Table 2). All the associations remained significant after FDR-correction except for the association with *P_T_* = 0.05. Associations between PRS for ADHD and numerical WM were also negative and nominally significant at all significance thresholds except at *P_T_* <0.01. Only the association between PRS for ADHD at *P_T_* <0.05 survived FDR-correction (*β* = −0.078, 95% CI = −0.131, −0.025; *p-uncorrec.* = 0.004, *p*-FDR = 0.028).

**Table 2.** Association results between polygenic risk scores (PRS) for attention-deficit hyperactivity disorder (ADHD) and autism spectrum disorder (ASD) with cognitive measures at baseline adjusting by age, sex and the first four genetic principal components

Outcome	PRS		*N*	Beta coefficient (95% CIs)	*p*-uncorrec.	*p*-FDR	Δ*R*^2^
Verbal WM (*d′*) (*n* = 1504)	ADHD	*P_T_ < 0.01*	1504	−0.08 (−0.129; −0.028)	0.003	0.018	0.005
		*P_T_ < 0.05*	1504	−0.058 (−0.108; −0.007)	0.026	0.105	0.003
		*P_T_ < 0.1*	1504	−0.077 (−0.128; −0.026)	0.003	0.018	0.005
		*P_T_ < 0.5*	1504	−0.08 (−0.131; −0.03)	0.002	0.018	0.006
		*P_T_ < 1*	1504	−0.081 (−0.132; −0.03)	0.002	0.018	0.006
	ASD	*P_T_ < 0.01*	1504	−0.01 (−0.062; 0.042)	0.701	0.980	0.000
		*P_T_ < 0.05*	1504	0.012 (−0.039; 0.063)	0.645	0.980	0.000
		*P_T_ < 0.1*	1504	0.02 (−0.03; 0.071)	0.431	0.980	0.000
		*P_T_ < 0.5*	1504	0.003 (−0.048; 0.054)	0.904	0.980	0.000
		*P_T_ < 1*	1504	0.002 (−0.049; 0.053)	0.941	0.980	0.000
Numerical WM (*d′*) (*n* = 1490)	ADHD	*P_T_ < 0.01*	1490	−0.039 (−0.089; 0.011)	0.124	0.371	0.001
		*P_T_ < 0.05*	1490	−0.076 (−0.127; −0.026)	0.003	0.018	0.006
		*P_T_ < 0.1*	1490	−0.06 (−0.111; −0.01)	0.019	0.096	0.004
		*P_T_ < 0.5*	1490	−0.055 (−0.105; −0.005)	0.033	0.110	0.003
		*P_T_ < 1*	1490	−0.057 (−0.107; −0.006)	0.028	0.105	0.003
	ASD	*P_T_ < 0.01*	1490	−0.001 (−0.052; 0.049)	0.963	0.980	0.000
		*P_T_ < 0.05*	1490	−0.012 (−0.062; 0.039)	0.653	0.980	0.000
		*P_T_ < 0.1*	1490	0.008 (−0.042; 0.058)	0.754	0.980	0.000
		*P_T_ < 0.5*	1490	−0.001 (−0.051; 0.05)	0.980	0.980	0.000
		*P_T_ < 1*	1490	0.006 (−0.045; 0.056)	0.829	0.980	0.000
HRTSE (*n* = 1487)	ADHD	*P_T_ < 0.01*	1487	0.400 (−3.742; 4.542)	0.850	0.980	0.000
		*P_T_ < 0.05*	1487	0.823 (−3.336; 4.981)	0.699	0.980	0.000
		*P_T_ < 0.1*	1487	−0.174 (−4.359; 4.010)	0.935	0.980	0.000
		*P_T_ < 0.5*	1487	−0.404 (−4572; 3.765)	0.850	0.980	0.000
		*P_T_ < 1*	1487	−0.453 (−4.625; 3.718)	0.832	0.980	0.000
	ASD	*P_T_ < 0.01*	1487	−2.044 (−6.245; 2.157)	0.341	0.931	0.001
		*P_T_ < 0.05*	1487	−1.597 (−5.734; 2.54)	0.450	0.980	0.001
		*P_T_ < 0.1*	1487	−0.114 (−4.245; 4.017)	0.957	0.980	0.000
		*P_T_ < 0.5*	1487	−0.057 (−4.181; 4.066)	0.978	0.980	0.000
		*P_T_ < 1*	1487	−0.231 (−4.367; 3.905)	0.913	0.980	0.000

NOTE: *P_T_*, significance threshold for inclusion of variants in the polygenic score; CI, confidence interval; *p*-uncorr., uncorrected *p* value; *p*-FDR, false discovery rate adjusted *p* value; Δ*R*^2^, difference between the *R*^2^ of the full model (PRS + covariates) compared to the *R*^2^ of the model including only covariates.

Polygenic risk for ASD did not show significant associations with WM measures. Although not significant, a consistent pattern of results was observed suggesting positive associations between PRS for ASD and verbal and numerical WM performance (Table 2).

No significant associations were found between PRS for ADHD or ASD and HRTSE (Table 2).

Significant interactions between PRS for ADHD and sex were observed on verbal (*p* value for interaction for *P_T_ <*0.5 and *P_T_ <*1 were 0.028 and 0.039, respectively) and numerical WM (*p* value for interaction for *P_T_ <*0.01 and *P_T_ <*0.05 were 0.038 and 0.028, respectively) indicating that negative associations between PRS for ADHD and WM measures were stronger among boys (online Supplementary Table S5). Stratified results revealed that significant associations were limited to boys (online Supplementary Table S5).

### Association between PRS and cognitive performance over 1-year period

No significant associations at *p*-FDR<0.05 were observed between PRS for ADHD or ASD and WM or HTRSE measures modeled as 1-year trajectories (Table 3).

**Table 3.** Association results between polygenic risk scores (PRS) for attention-deficit hyperactivity disorder (ADHD) and autism spectrum disorder (ASD) with cognitive 1-year trajectories adjusting by age, sex and the first four genetic principal components

Outcome	PRS		*N*	Beta coefficient (95% CIs)	*p*-uncorrec.	*p*-FDR	Δ*R*^2^
Verbal WM (*d′*)	ADHD	*P_T_ < 0.01*	4684	0.005 (−0.032; 0.042)	0.786	0.870	0.00001
		*P_T_ < 0.05*	4684	0.002 (−0.034; 0.039)	0.899	0.916	0.00000
		*P_T_ < 0.1*	4684	−0.006 (−0.043; 0.031)	0.751	0.867	0.00003
		*P_T_ < 0.5*	4684	−0.008 (−0.045; 0.029)	0.658	0.867	0.00006
		*P_T_ < 1*	4684	−0.007 (−0.044; 0.031)	0.729	0.867	0.00003
	ASD	*P_T_ < 0.01*	4684	0.007 (−0.031; 0.044)	0.724	0.867	0.00003
		*P_T_ < 0.05*	4684	0.024 (−0.013; 0.06)	0.209	0.722	0.00045
		*P_T_ < 0.1*	4684	0.021 (−0.016; 0.058)	0.265	0.722	0.00035
		*P_T_ < 0.5*	4684	0.036 (−0.001; 0.072)	0.056	0.722	0.00113
		*P_T_ < 1*	4684	0.041 (0.004; 0.077)	0.030	0.722	0.00146
Numerical WM (*d′*)	ADHD	*P_T_ < 0.01*	4668	0.008 (−0.029; 0.045)	0.680	0.867	0.00003
		*P_T_ < 0.05*	4668	0.016 (−0.021; 0.053)	0.398	0.859	0.00017
		*P_T_ < 0.1*	4668	0.014 (−0.023; 0.051)	0.470	0.859	0.00013
		*P_T_ < 0.5*	4668	0.01 (−0.027; 0.048)	0.588	0.859	0.00006
		*P_T_ < 1*	4668	0.01 (−0.027; 0.047)	0.602	0.859	0.00006
	ASD	*P_T_ < 0.01*	4668	0.015 (−0.022; 0.053)	0.418	0.859	0.00019
		*P_T_ < 0.05*	4668	0.024 (−0.013; 0.061)	0.206	0.722	0.00047
		*P_T_ < 0.1*	4668	0.022 (−0.015; 0.059)	0.249	0.722	0.00039
		*P_T_ < 0.5*	4668	0.029 (−0.008; 0.066)	0.128	0.722	0.00072
		*P_T_ < 1*	4668	0.031 (−0.006; 0.068)	0.097	0.722	0.00085
HRTSE	ADHD	*P_T_ < 0.01*	4646	0.886 (−3.72; 4.56)	0.595	0.859	0.00017
		*P_T_ < 0.05*	4646	2.872 (−2.1; 6.161)	0.081	0.722	0.00150
		*P_T_ < 0.1*	4646	2.105 (−2.211; 6.066)	0.205	0.722	0.00085
		*P_T_ < 0.5*	4646	1.723 (−1.924; 6.407)	0.304	0.759	0.00060
		*P_T_ < 1*	4646	1.986 (−2.202; 6.113)	0.236	0.722	0.00075
	ASD	*P_T_ < 0.01*	4646	0.95 (−7.558; 0.522)	0.565	0.859	0.00007
		*P_T_ < 0.05*	4646	0.173 (−7.078; 1.2)	0.916	0.916	−0.00002
		*P_T_ < 0.1*	4646	0.394 (−6.066; 2.099)	0.812	0.870	−0.00001
		*P_T_ < 0.5*	4646	−0.917 (−4.955; 3.117)	0.577	0.859	0.00013
		*P_T_ < 1*	4646	−1.105 (−5.364; 2.764)	0.501	0.859	0.00018

NOTE: *P_T_*, significance threshold for inclusion of variants in the polygenic score; CI, confidence interval; *p*-uncorr., uncorrected *p* value; *p*-FDR, false discovery rate adjusted *p* value; the *R*^2^ of the full model (PRS + covariates) compared to the *R*^2^ of the model including only covariates.

A nominally significant association was observed between PRS for ASD at *P_T_* < 1 and verbal WM (*β* = 0.041, 95% CI = 0.004, 0.077; *p-uncorrec.* = 0.032, *p*-FDR = 0.497) suggesting that higher polygenic load for ASD is related to increased cognitive growth over the 1-year period (online Supplementary Fig. S3). Individuals with higher polygenic risk for ASD would improve faster than individuals with lower loads.

No significant interactions by sex were detected (online Supplementary Table S6).

### Sensitivity analysis

Similar results were observed after excluding 174 children with an ADHD diagnosis (online Supplementary Table S6). Specifically, PRS for ADHD were negatively nominally associated with verbal WM performance (*d′*) assessed at baseline across all significance thresholds. At all significance thresholds, these associations remained significant following FDR-correction except at *P_T_*  < 0.05. The strongest association was observed at *P_T_* = 0.05 (*β* = −0.086, 95% CI = −0.139, −0.033; *p-uncorrec.* = 0.002, *p*-FDR = 0.026). Nominally significant associations were also observed with numerical WM performance (*d′*) for all significance thresholds except *P_T_* < 0.01*_._* Of these, one association survived FDR-correction (*β* = −0.078, 95% CI = −0.131, −0.025; *p-uncorrec.* = 0.004, *p*-FDR = 0.028) (online Supplementary Table S7).

As previously observed in the sample including children with ADHD diagnosis, PRS for ASD did not show significant associations with WM measures and no significant associations were observed between PRS for ADHD or ASD and HRTSE (online Supplementary Table S7).

## Discussion

In the current study, we examined the genetic overlap between ADHD and ASD and cognitive abilities among school-aged children from the general population. We found that polygenic risk for ADHD was associated with worse WM performance. These associations were observed at baseline assessments but not over time. In contrast, PRS for ASD did not show significant associations with any of the cognitive measures analyzed at baseline. Only a nominal association was observed between PRS for ASD and increased cognitive growth in verbal WM over time, although this association did not survive FDR-correction. The exclusion of individuals diagnosed with ADHD from the analyses did not change meaningfully these findings. No associations were observed for HRTSE.

As expected, the polygenic risk for ADHD was positively associated with ADHD symptoms total score, inattention and hyperactivity. The association between polygenic risk for ADHD and hyperactivity and inattention was very similar in strength and magnitude. These findings provide additional support for a dimensional conceptualization of ADHD with genetic factors operating through the distribution of the trait (Larsson, Anckarsater, Råstam, Chang, & Lichtenstein, 2012).

In our study, PRS for ADHD were associated with worse baseline WM performance which is in line with previous studies reporting negative associations between polygenic risk for ADHD and cognitive measures. In the Avon Longitudinal Study of Parents And Children population-based birth cohort, negative associations were found between the PRS for ADHD and intelligence and WM (*N* = 6832) (Martin et al., 2015). Also, PRS for ADHD were related with worse educational outcomes (lower scores obtained in national examinations) among children (*N* = 5748) and low intelligence quotient (IQ) scores among adolescents (*N* = 4958) from the general population (Stergiakouli et al., 2017). Another study conducted in three cohorts, the Generation Scotland Scottish Family Health Study (*N* = 9863), the Lothian Birth Cohorts 1936 and 1921 (*N* = 1522), and the Brisbane Adolescent Twin Sample (BATS) (*N* = 921), did not show consistent results regarding the associations between polygenic risk for ADHD and cognitive outcomes, but a negative association was observed with IQ at age 11 years in the Lothian Birth Cohorts (Clarke et al., 2016). A more recent study based on a large sample of adult individuals drawn from the UK Biobank Study (*N* = 135 726) found that verbal-numerical reasoning scores, a cognitive measurement related to IQ, decreased with increasing polygenic load for ADHD (Du Rietz et al., 2018). Remarkably, in this last study, summary statistics used to compute PRS were the same used in the current study. Therefore, our findings extend previous results suggesting a genetic overlap between ADHD and worse WM performance during childhood.

Interestingly, when stratifying by sex, we observed that the detrimental effects of ADHD polygenic risk on WM performance at baseline were limited to boys. This is in contrast with previous studies that did not find evidence for differential sex-effects (Du Rietz et al., 2018; Stergiakouli et al., 2017). Another study observed sex-differential effects for PRS for ADHD on WM assessed at age 8 years but limited to girls (Martin et al., 2015). Our results may be explained by the higher prevalence of ADHD symptoms observed among boys in our sample, which is in agreement with the literature (Polanczyk et al., 2007). Although the reasons underlying the higher rates of childhood ADHD among boys remain unclear, recent research indicates that the genetic variants related to ADHD largely overlap between boys and girls (Martin et al., 2018). Nevertheless, an alternative explanation for our findings regards the possibility that genetic susceptibility for ADHD indexed using PRS operates via different mechanisms in boys and girls.

Considering all the above findings, genetic risk variants for ADHD may have pleiotropic effects in closely related cognitive domains. These pleiotropic effects may arise from common genetic variants influencing both ADHD symptoms and cognition, reflecting horizontal or biological pleiotropy; or genetically influenced mechanisms may lead to one trait that influences the other, also known as vertical or mediated pleiotropy (Hemani, Bowden, & Davey Smith, 2019). Furthermore, detecting these associations in population-based samples, even after excluding clinical cases, suggest that the association between genetic risk for ADHD and worse WM performance is independent of clinical status.

To our knowledge, this study is the first study to examine the association between polygenic risk for ADHD and ASD on 1-year cognitive-developmental trajectories. The interest in studying 1-year trajectories was exploring whether polygenic risk for ADHD and ASD was related to cognitive changes over this period. While we have observed that overall all children improve their performance from the first to the last time that they complete the task, there is substantial interindividual variation in the speed (whether improvement occurs in the first or last visits) and magnitude (difference in the score from first to the last visit) of this improvement (López-Vicente et al., 2016; Suades-González et al., 2017). However, our significant findings were largely limited to baseline assessments of WM performance. This is largely in line with a previous study conducted in the entire BREATHE sample (*N* = 2897), reporting worse WM performance at baseline but similar cognitive growth trajectories for individuals with and without ADHD diagnosis for tasks with numbers as stimuli (López-Vicente et al., 2016). However, López-Vicente et al. observed differences in WM trajectories for verbal tasks between children with and without an ADHD diagnosis. Our results suggest that genetic risk variants for ADHD may be underlying the observed worse WM performance at baseline, but they are not related to variability in 1-year cognitive-developmental trajectories. In other words, in our study, children with a higher genetic risk for ADHD showed worse performance the first time they were tested, but these variants do not influence the subsequent improvement in WM development during 1-year follow-up. Although we cannot rule out practice effects (Mollica et al., 2005), the improvement in WM tasks through childhood and adolescence is thought to be an age-dependent process underlined by brain maturation (Moriguchi et al., 2015; Tamnes et al., 2013; Vuontela et al., 2003).

Regarding HRTSE the lack of significant associations with this domain may be related to findings indicating that PRS for ADHD are more strongly related with hyperactivity than inattention symptoms (Brikell et al., 2020; Martin, Hamshere, Stergiakouli, O'Donovan, & Thapar, 2014; Sudre et al., 2019; Vuijk et al., 2019). However, this was not the case for our sample where we found similar associations between PRS for ADHD and hyperactivity and inattention symptoms. Indeed, as it has been previously stated (Sudre et al., 2019), WM and other cognitive abilities such as intelligence have been shown to be genetically correlated with ADHD (Martin et al., 2014; Savage et al., 2018). However, up to our knowledge, genetic correlation or overlap between ADHD and attentional measures at a molecular level remains to be established.

In our study, the polygenic risk of ASD was not significantly associated with any of the cognitive measures analyzed. Only a nominal association was observed between polygenic for ASD and increased cognitive growth in verbal WM over time suggesting that common genetic variants related to ASD may be related to better cognitive performance on this domain. This agrees with previous findings from the abovementioned studies by Clarke and colleagues in three cohorts and findings in one of the samples analyzed in Schork and colleagues (Clarke et al., 2016; Schork et al., 2019). In these studies, a higher polygenic risk for ASD was associated with better cognitive functioning. Thus, although not significant, our results at baseline and over time show a direction consistent with previous findings. The fact that they were not significant may not be only related to sample size since this association was significant in a smaller sample in the study by Schork et al. (2019). Further research is needed to elucidate which are the effects of genetic variants related to ASD on cognition.

Our results should be interpreted in the context of several strengths and limitations. One of the strengths of the study regards the obtention of cognitive measures using computerized tests which reduces the examiner bias. Also, we used well-powered GWAS to compute PRS, a key aspect to increase the accuracy of the PRS (Choi et al. 2020). Among the limitations, first, our study has a relatively small sample (*N* = 1667). It has been pointed out that a target sample of around 2000 subjects provides sufficient power to detect a significant proportion of variance explained, which is slightly larger than our sample (Marees, Stringer, Claire, & Derks, 2018). Second, ASD symptoms were not available, thus we were not able to confirm that PRS for ASD were capturing this phenotype in our sample. Third, children included in the study presented better cognitive performance compared to excluded children which might have resulted in underestimated associations between PRS for ADHD and cognition. Finally, polygenic scores typically explain only a small proportion of the total phenotypic variance of complex traits (Wray et al., 2014).

To conclude, common genetic variants related to ADHD may be, at least partially, underlying cognitive deficits among school-aged children regarding WM performance. These associations were limited to baseline assessment suggesting that polygenic risk for ADHD is not associated with 1-year trajectories of cognitive development. Our findings suggest that genetic risk variants for ADHD may have pleiotropic effects in WM, a closely related neurodevelopmental domain in the context of ADHD. Further research is needed to characterize the neurobiological mechanisms underlying the genetic overlap between ADHD and WM.

## Supplementary material

For supplementary material accompanying this paper visit https://doi.org/10.1017/S0033291720003189. click here to view supplementary material
