# Supplementary material for: Polygenic risk for ADHD and ASD and their relation with cognitive measures in school children
Source: Psychol Med. 2020 Sep 14;52(7):1356–64. doi: 10.1017/S0033291720003189 (PMC9157306; doi:10.1017/S0033291720003189)
Supplement: Supplementary file 1 [file S0033291720003189sup001.docx]

**Supplementary Material**

**Title: Polygenic Risk for ADHD and ASD and their relation with cognitive measures in school children**

**Authors:** Sofia Aguilar-Lacasaña, Natàlia Vilor-Tejedor, Philip Jansen, Mònica López-Vicente, Mariona Bustamante, Miguel Burgaleta, Jordi Sunyer, Silvia Alemany

Table of Contents

[**Methods S1.** Assessment of inattentiveness using the ANT 2](#_Toc45019809)

[**Methods S2.** Assessment of working memory performance using n-back task 3](#_Toc45019810)

[**Methods S3.** Genotyping 4](#_Toc45019811)

[**Table S1**. Number of variants included in the polygenic risk scores for each p-value threshold (P_T_). 5](#_Toc45019812)

[**Table S2.** Characteristics of the sample. 6](#_Toc45019813)

[**Table S3.** Characteristics of the sample by sex. 7](#_Toc45019814)

[**Figure S1.** Pearson correlations between cognitive measurements including verbal and numerical working memory (WM) and HRTSE (N=1,257). Coloured squares indicate significant associations (p < 0.05). 8](#_Toc45019815)

[**Figure S2.** Pearson correlations between PRS for ADHD and ASD for all significance thresholds (N=1,667). Coloured squares indicate significant associations (p < 0.05). 9](#_Toc45019816)

[**Table S4**. Validation analysis testing associations between polygenic risk scores (PRS) for attention-deficit hyperactivity disorder (PRS-ADHD) with behavioural measurements of ADHD symptoms (N=1,555) adjusting by age, sex and the first four genetic principal components. 10](#_Toc45019817)

[**Table S5**. Stratified results by sex for association results between polygenic risk scores (PRS) for attention-deficit hyperactivity disorder (ADHD) and autism spectrum disorder (ASD) with cognitive measures at baseline adjusting by age and the first four genetic principal components. P-value of the interaction by each PRS and sex is indicated. 11](#_Toc45019818)

[**Figure S3.** Polygenic risk score (PRS) for ASD at PT<1 and developmental trajectory of verbal working memory (WM). 13](#_Toc45019819)

[**Table S6**. Interaction effects between polygenic risk scores (PRS) for attention-deficit hyperactivity disorder (ADHD) and autism spectrum disorder (ASD) and sex on cognitive 1-year trajectories adjusting by age and the first four genetic principal components. 14](#_Toc45019820)

[**Table S7**. Sensitivity analysis testing the association results between polygenic risk scores (PRS) for attention-deficit hyperactivity disorder (ADHD) and autism spectrum disorder (ASD) and cognitive outcomes including HRTSE (N=1,487), verbal (N=1,330) and numerical WM performance (N= 1,316), excluding 174 children diagnosed with ADHD. All analyses were adjusted by age, sex and the first four genetic principal components. 16](#_Toc45019821)

[**References** 18](#_Toc45019822)

### **Methods S1.** Assessment of inattentiveness using the ANT

The ANT (Rueda et al., 2004) takes approximately 10 minutes to complete. In this task, a row of five yellow fish appears either above or below a fixation point is presented. Children were invited to “feed” the central fish as quickly as possible by pressing either the right or the left button on the mouse depending on the direction in which the fish in the middle was pointing while ignoring the flanker fish, which pointed in either the same (congruent) or opposite (incongruent) direction as to the middle fish. The target was preceded by visual signals that informed either about the upcoming of the target only (alerting cue) or about the upcoming of the target as well as its location (orienting cue). Reaction times (i.e.,time between presenting a stimulus and the reaction of the participant to that stimulus) were used to calculate different outcome measures. Among those outcome measures, we analyzed the hit reaction time standard error (HRTSE) (standard error of reaction time for correct responses) as a measure of inattentiveness.

Higher HRT-SE scores indicate reduced executive and attentional resources, and are characteristic of the performance in patients with ADHD (Bellgrove, Hester, & Garavan, 2004; MacDonald, Nyberg, & Bäckman, 2006).

### **Methods S2.** Assessment of working memory performance using n-back task

In the n-back task, the subject was required to monitor a series of stimuli presented in the center of the screen, and to respond whenever a stimulus matched the one presented an n-number of trials previously (n=1, 2, or 3), which are defined as loads. Higher loads imply higher demands on WM. Participants completed three blocks (1-, 2-, and 3-back) for different stimulus including: colors, letters, numbers, and words. Here, we used numbers and words as stimuli in the 3-back level. This load was chosen because it predicts general mental abilities (Shelton, Elliott, & Matthews, 2011). We selected numbers and words as stimuli because they showed a clear age-dependent slope in the four measures. We obtained various measures for each trial, including accuracy measures (hits, correct rejections, false alarms, and misses) and hit reaction time (HRT, recorded when the participant correctly identified a target). We calculated d prime (*d′*), a widely used outcome of WM, which is derived from signal detection theory and allows to distinguish between signal and noise (Haatveit et al., 2010; Wickens, 2002). The value of *d′* is computed as z (hit rate) − z (false alarm rate), with higher *d′* indicating better signal detection and more accurate performance. This score incorporates more information about WM capacity than HRT and it has been suggested to be more sensitive to interindividual variability (Forns et al., 2014).

### **Methods S3.** Genotyping

From the 2,897 children participating in the original BREATHE cohort, 2,492 (86%) accepted to provide saliva for DNA genotyping. Saliva samples were collected using the Oragene DNA OG-500kit (DNA Genotek). From these children with available saliva samples, a final subset of 1,778 (61%) children was selected for genome-wide genotyping after applying a filtering criterion. Filtering criteria included low quality DNA (n=64 exclusions), adopted children (n=34 exclusions), siblings or twins (n=92 exclusions), being born outside Europe or having parents born outside Europe (n=482 exclusions), and no data available on residential address (n=42 exclusions).

Genome-wide genotyping was performed using the HumanCore BeadChipWG-330-1,101 (Illumina). Genotypes were called using the GeneTrain2.0 algorithm (with a default threshold of 0.15) based on HapMap clusters implemented in the GenomeStudio software. The human version was Human genome 19 (hg19) Genome Reference Consortium Human Build 37 (GRCh37). PLINK was used to perform genotyping quality control (Purcell et al. 2007). The final discovery genetic data set included 240,103 genetic variants for 1,667 individuals.

### **Table S1**. Number of variants included in the polygenic risk scores for each p-value threshold (P_T_).

|  | Trait | |
| --- | --- | --- |
|  | **ADHD** | **ASD** |
| *P_T_ < 0.01* | 2,863 | 1,778 |
| *P_T_ < 0.05* | 8,894 | 6,935 |
| *P_T_ < 0.1* | 14,472 | 12,048 |
| *P_T_ < 0.5* | 41,863 | 39,204 |
| *P_T_ < 1* | 59,284 | 56,131 |

# Table S2. Characteristics of the sample.

| ***Characteristic*** | **Excluded (no genetic data available)**  **(n=1230)** | **Included**  **(n=1667)** | **P-value^a^** |
| --- | --- | --- | --- |
| **Sex (female), n (%)** | 643 (52.3) | 782 (46.9) | 0.004 |
| **Age (years), mean (SD)** | 8.6 (0.9) | 8.5 (0.9) | 0.107 |
| ***Cognitive measures*** | | | |
| **Verbal WM, mean (SD)^b^** | 1.2 (1.0) | 1.4 (1.0) | <0.001 |
| **Numerical WM, mean (SD) ^b^** | 1.1 (1.0) | 1.3 (1.0) | <0.001 |
| **HRTSE, mean (SD)** | 279.4 (94.5) | 267.2 (88.0) | 0.001 |
| ***Behavioral measures*** | | | |
| **ADHD symptom scores, mean (SD)** | 8.7 (9.7) | 7.7 (9.4) | 0.001 |
| **Inattention, mean (SD)** | 5.5 (5.9) | 4.6 (5.6) | <0.001 |
| **Hyperactivity, mean (SD)** | 3.2 (5.0) | 3.1 (4.9) | 0.597 |
| NOTE: ADHD Total Symptoms score, teacher-reported attention deficit hyperactivity disorder symptoms where higher scores indicate more symptoms. HRTSE, standard error of the hit reaction time obtained from the Attentional Network Test, higher scores indicate more inattentiveness. WM, working memory performance (d’ values) from the 3-back task of the n-back test with words (Verbal) and numbers (Numerical) as stimuli, higher values indicate better WM.  ^a^Analysis of differences between included and excluded groups were conducted using independent-samples t-test for continuous variables and χ2 tests for categorical variables.  ^b^Baseline (visit 1) | | | |

| **Characteristic** | **Girls**  **(N=782)** | **Boys**  **(N=863)** | **P-value^a^** |
| --- | --- | --- | --- |
| **Age (years), mean (SD)** | 8.6 (0.9) | 8.5 (0.9) | 0.345 |
| **Cognitive measures** |  |  |  |
| **Verbal WM, mean (SD)^a^** | 1.3 (1.0) | 1.3 (1.0) | 0.241 |
| **Numerical WM, mean (SD)^a^** | 1.1 (1.0) | 1.2 (1.0) | 0.030 |
| **HRTSE, mean (SD)** | 285.3 (89.9) | 259.9 (90.4) | <0.001 |
| **Behavioral measures** |  |  |  |
| **ADHD symptom scores, mean (SD)** | 5.7 (7.7) | 10.5 (10.6) | <0.001 |
| **Inattention, mean (SD)** | 3.7 (5.0) | 6.3 (6.1) | <0.001 |
| **Hyperactivity, mean (SD)** | 2.0 (3.6) | 4.3 (5.7) | <0.001 |
| NOTE: ADHD Total Symptoms score, teacher-reported attention deficit hyperactivity disorder symptoms where higher scores indicate more symptoms. HRTSE, standard error of the hit reaction time obtained from the Attentional Network Test, higher scores indicate worse attention performance. WM, working memory performance (d’ values) from the 3-back task of the n-back test with words (Verbal) and numbers (Numerical) as stimuli, higher values indicate better WM.  ^a^Baseline (visit 1) | | | |

# Table S3. Characteristics of the sample by sex.

***
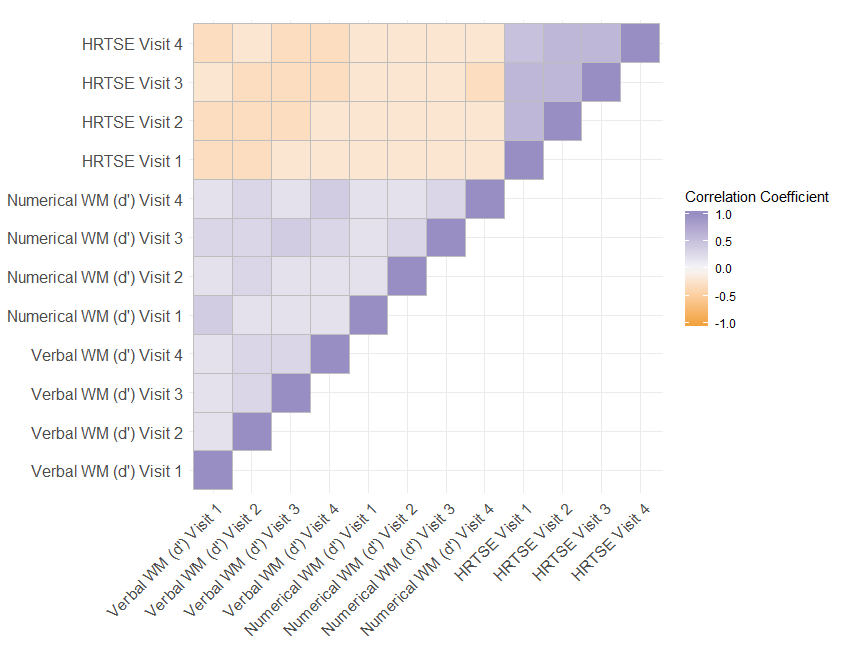
***

### **Figure S1.** Pearson correlations between cognitive measurements including verbal and numerical working memory (WM) and HRTSE (N=1,257). Coloured squares indicate significant associations (p < 0.05).

**
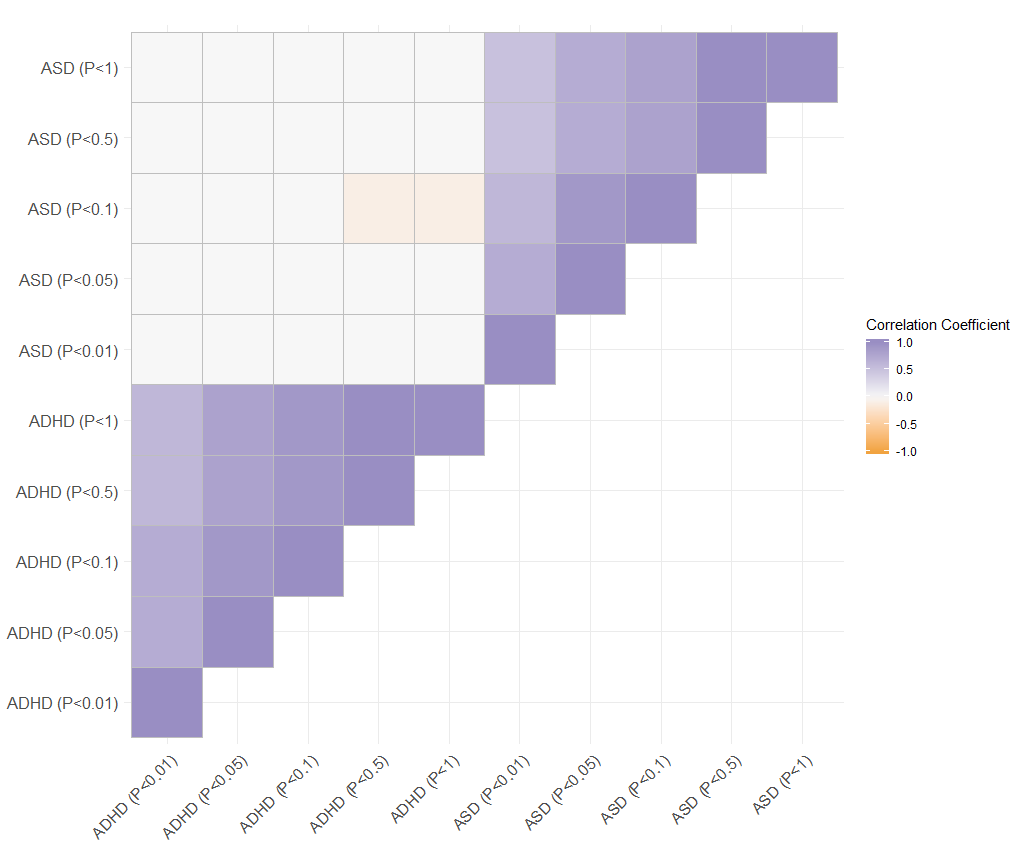
**

### **Figure S2.** Pearson correlations between PRS for ADHD and ASD for all significance thresholds (N=1,667). Coloured squares indicate significant associations (p < 0.05).

### **Table S4**. Validation analysis testing associations between polygenic risk scores (PRS) for attention-deficit hyperactivity disorder (PRS-ADHD) with behavioural measurements of ADHD symptoms (N=1,555) adjusting by age, sex and the first four genetic principal components.

| **Outcome** | **PRS-ADHD** | **N** | **Beta coefficient (95% CIs)** | **P-value** | **ΔR^2^** |
| --- | --- | --- | --- | --- | --- |
| **ADHD symptoms total score** | *P_T_ < 0.01* | 1602 | 0.516 (0.067, 0.965) | 0.025 | 0.003 |
|  | *P_T_ < 0.05* | 1602 | 0.646 (0.196, 1.096) | 0.005 | 0.005 |
|  | *P_T_ < 0.1* | 1602 | 0.739 (0.289, 1.188) | 0.001 | 0.006 |
|  | *P_T_ < 0.5* | 1602 | 0.804 (0.356, 1.252) | 0.000 | 0.007 |
|  | *P_T_ < 1* | 1602 | 0.773 (0.325, 1.221) | 0.001 | 0.007 |
| **Inattention** | *P_T_ < 0.01* | 1614 | 0.216 (-0.052, 0.484) | 0.115 | 0.001 |
|  | *P_T_ < 0.05* | 1614 | 0.319 (0.05, 0.587) | 0.020 | 0.003 |
|  | *P_T_ < 0.1* | 1614 | 0.368 (0.099, 0.637) | 0.008 | 0.004 |
|  | *P_T_ < 0.5* | 1614 | 0.414 (0.147, 0.682) | 0.003 | 0.005 |
|  | *P_T_ < 1* | 1614 | 0.386 (0.118, 0.654) | 0.005 | 0.005 |
| **Hyperactivity** | *P_T_ < 0.01* | 1614 | 0.24 (0.005, 0.476) | 0.046 | 0.002 |
|  | *P_T_ < 0.05* | 1614 | 0.268 (0.033, 0.504) | 0.026 | 0.003 |
|  | *P_T_ < 0.1* | 1614 | 0.319 (0.083, 0.555) | 0.008 | 0.004 |
|  | *P_T_ < 0.5* | 1614 | 0.36 (0.125, 0.595) | 0.003 | 0.005 |
|  | *P_T_ < 1* | 1614 | 0.359 (0.124, 0.595) | 0.003 | 0.005 |
| NOTE: *P_T_*, significance threshold for inclusion of variants in the polygenic score; CI; Confidence Interval; P-value, uncorrected P-value; difference between the R² of the full model (PRS + covariates) compared to the R² of the model including only covariates. | | | | | |
|  | | | | | |

### **Table S5**. Stratified results by sex for association results between polygenic risk scores (PRS) for attention-deficit hyperactivity disorder (ADHD) and autism spectrum disorder (ASD) with cognitive measures at baseline adjusting by age and the first four genetic principal components. P-value of the interaction by each PRS and sex is indicated.

|  |  | | **FEMALES** | | | | **MALES** | | | | |
| --- | --- | --- | --- | --- | --- | --- | --- | --- | --- | --- | --- |
| **Outcome** | **PRS** | | **N** | **Beta coefficient (95% CIs)** | **P-value** | **ΔR^2^** | **N** | **Beta coefficient (95% CIs)** | **P-value** | **ΔR^2^** | **P-value interaction** |
| **Verbal WM (*d'*)** | **ADHD** | *P_T_ < 0.01* | 703 | -0.032 (-0.108; 0.044) | 0.408 | 0,0009 | **801** | **-0.115 (-0.183; -0.047)** | **0.001** | 0,0128 | 0.102 |
|  |  | *P_T_ < 0.05* | 703 | -0.005 (-0.079; 0.069) | 0.898 | 0,0000 | **801** | **-0.104 (-0.174; -0.035)** | **0.004** | 0,0101 | 0.053 |
|  |  | *P_T_ < 0.1* | 703 | -0.036 (-0.108; 0.037) | 0.334 | 0,0013 | **801** | **-0.12 (-0.191; -0.048)** | **0.001** | 0,0126 | 0.101 |
|  |  | *P_T_ < 0.5* | 703 | -0.02 (-0.092; 0.052) | 0.582 | 0,0004 | **801** | **-0.135 (-0.205; -0.064)** | **0.000** | 0,0165 | **0.028** |
|  |  | *P_T_ < 1* | 703 | -0.024 (-0.096; 0.049) | 0.527 | 0,0005 | **801** | **-0.132 (-0.202; -0.062)** | **0.000** | 0,0159 | **0.039** |
|  | **ASD** | *P_T_ < 0.01* | 703 | 0.018 (-0.054; 0.091) | 0.622 | 0,0003 | 801 | -0.034 (-0.108; 0.04) | 0.363 | 0,0010 | 0.262 |
|  |  | *P_T_ < 0.05* | 703 | 0.022 (-0.048; 0.092) | 0.535 | 0,0005 | 801 | 0.004 (-0.07; 0.077) | 0.922 | 0,0000 | 0.587 |
|  |  | *P_T_ < 0.1* | 703 | 0.016 (-0.054; 0.086) | 0.653 | 0,0003 | 801 | 0.026 (-0.047; 0.099) | 0.487 | 0,0006 | 0.935 |
|  |  | *P_T_ < 0.5* | 703 | -0.009 (-0.079; 0.061) | 0.805 | 0,0001 | 801 | 0.016 (-0.058; 0.089) | 0.676 | 0,0002 | 0.728 |
|  |  | *P_T_ < 1* | 703 | -0.011 (-0.081; 0.058) | 0.749 | 0,0001 | 801 | 0.015 (-0.059; 0.089) | 0.690 | 0,0002 | 0.716 |
| **Numerical WM (*d'*)** | **ADHD** | *P_T_ < 0.01* | 698 | 0.018 (-0.056; 0.093) | 0.635 | 0,0003 | **792** | **-0.088 (-0.156; -0.021)** | **0.011** | 0,0082 | **0.038** |
|  |  | *P_T_ < 0.05* | 698 | -0.016 (-0.09; 0.058) | 0.671 | 0,0003 | **792** | **-0.133 (-0.202; -0.064)** | **0.000** | 0,0177 | **0.028** |
|  |  | *P_T_ < 0.1* | 698 | -0.018 (-0.09; 0.054) | 0.621 | 0,0003 | **792** | **-0.106 (-0.177; -0.035)** | **0.004** | 0,0106 | 0.109 |
|  |  | *P_T_ < 0.5* | 698 | -0.007 (-0.079; 0.065) | 0.849 | 0,0001 | **792** | **-0.101 (-0.171; -0.031)** | **0.005** | 0,0099 | 0.087 |
|  |  | *P_T_ < 1* | 698 | -0.011 (-0.084; 0.061) | 0.766 | 0,0001 | **792** | **-0.099 (-0.169; -0.03)** | **0.005** | 0,0098 | 0.113 |
|  | **ASD** | *P_T_ < 0.01* | 698 | 0.031 (-0.04; 0.101) | 0.396 | 0,0010 | 792 | -0.036 (-0.109; 0.036) | 0.329 | 0,0012 | 0.095 |
|  |  | *P_T_ < 0.05* | 698 | 0.013 (-0.057; 0.082) | 0.721 | 0,0002 | 792 | -0.038 (-0.11; 0.035) | 0.308 | 0,0013 | 0.232 |
|  |  | *P_T_ < 0.1* | 698 | 0.033 (-0.036; 0.102) | 0.353 | 0,0012 | 792 | -0.017 (-0.09; 0.055) | 0.643 | 0,0003 | 0.225 |
|  |  | *P_T_ < 0.5* | 698 | 0.031 (-0.038; 0.1) | 0.378 | 0,0011 | 792 | -0.029 (-0.102; 0.043) | 0.433 | 0,0008 | 0.175 |
|  |  | *P_T_ < 1* | 698 | 0.041 (-0.029; 0.11) | 0.254 | 0,0019 | 792 | -0.025 (-0.098; 0.047) | 0.494 | 0,0006 | 0.143 |
| **HRTSE** | **ADHD** | *P_T_ < 0.01* | 701 | 0.684 (-5.775; 7.143) | 0.836 | 0,0001 | 786 | 0.756 (-4.681; 6.192) | 0.786 | 0,0001 | 0.969 |
|  |  | *P_T_ < 0.05* | 701 | 2.217 (-4.113; 8.547) | 0.494 | 0,0005 | 786 | -0.304 (-5.862; 5.254) | 0.915 | 0,0000 | 0.558 |
|  |  | *P_T_ < 0.1* | 701 | 1.967 (-4.217; 8.151) | 0.534 | 0,0005 | 786 | -2.103 (-7.837; 3.63) | 0.474 | 0,0006 | 0.347 |
|  |  | *P_T_ < 0.5* | 701 | 0.821 (-5.339; 6.981) | 0.795 | 0,0001 | 786 | -1.608 (-7.278; 4.062) | 0.580 | 0,0003 | 0.584 |
|  |  | *P_T_ < 1* | 701 | 1.103 (-5.122; 7.327) | 0.729 | 0,0001 | 786 | -1.917 (-7.541; 3.708) | 0.506 | 0,0005 | 0.495 |
|  | **ASD** | *P_T_ < 0.01* | 701 | -1.865 (-7.991; 4.261) | 0.552 | 0,0008 | 786 | -2.077 (-7.888; 3.735) | 0.485 | 0,0007 | 0.873 |
|  |  | *P_T_ < 0.05* | 701 | -1.094 (-7.046; 4.857) | 0.719 | 0,0003 | 786 | -2.043 (-7.821; 3.735) | 0.490 | 0,0007 | 0.753 |
|  |  | *P_T_ < 0.1* | 701 | -0.224 (-6.183; 5.736) | 0.942 | 0,0000 | 786 | -0.165 (-5.927; 5.598) | 0.955 | 0,0000 | 0.949 |
|  |  | *P_T_ < 0.5* | 701 | 0.803 (-5.153; 6.76) | 0.792 | 0,0001 | 786 | -1.111 (-6.867; 4.645) | 0.706 | 0,0002 | 0.605 |
|  |  | *P_T_ < 1* | 701 | 0.908 (-5.065; 6.882) | 0.767 | 0,0001 | 786 | -1.513 (-7.286; 4.259) | 0.609 | 0,0004 | 0.524 |
| NOTE: *P_T_*, significance threshold for inclusion of variants in the polygenic score; CI, Confidence Interval; P-uncorr., uncorrected P-value; P-FDR, false discovery rate adjusted *p*-value; ΔR^2^, difference between the R² of the full model (PRS + covariates) compared to the R² of the model including only covariates. | | | | | | | | | | | |


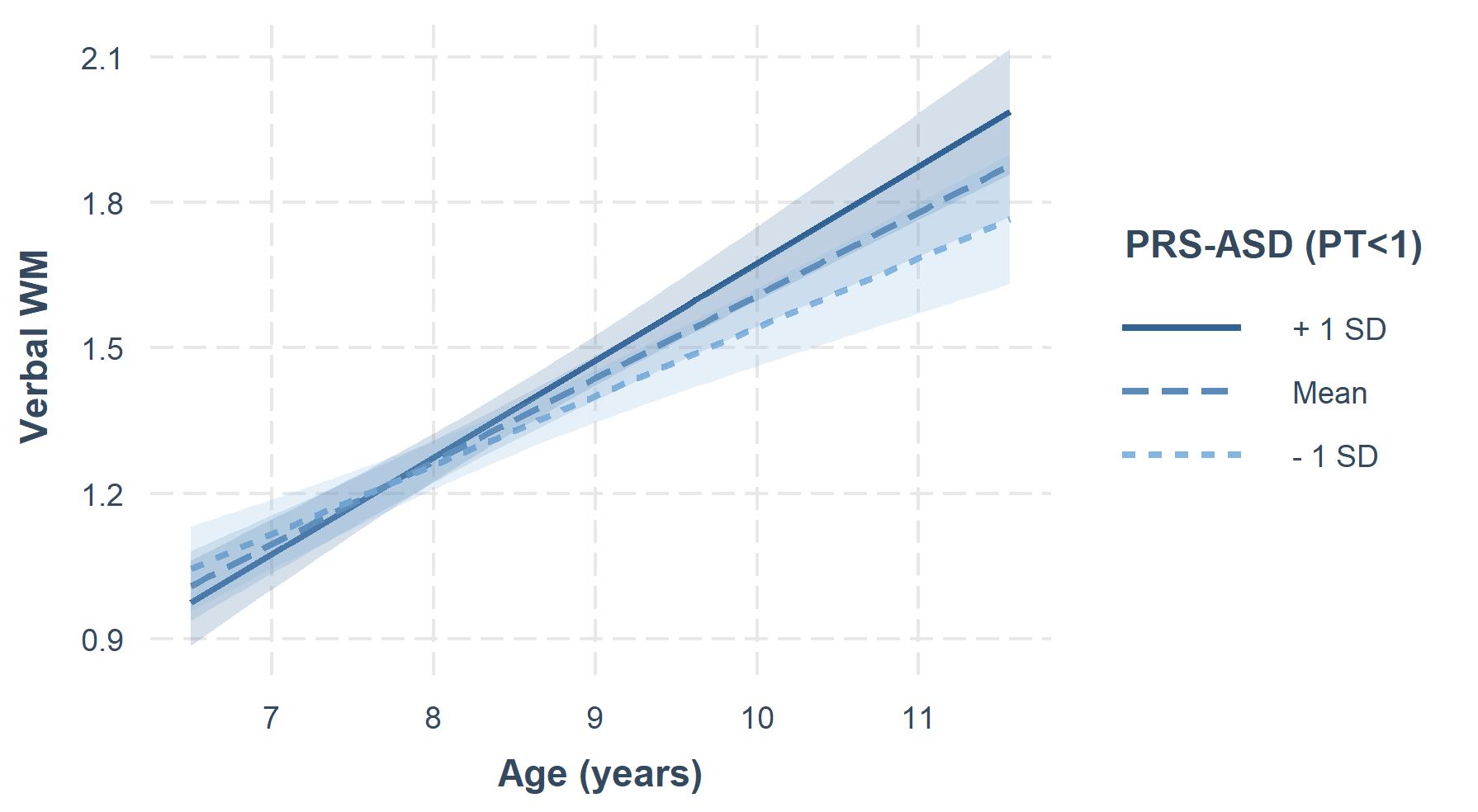


# Figure S3. Polygenic risk score (PRS) for ASD at PT<1 and developmental trajectory of verbal working memory (WM).

Graphical representation of the interaction between polygenic risk score (PRS) for ASD at PT<1 and age used to model 1-year trajectories of verbal WM. The slope of the dashed dark blue line depicts the change in WM development as a function of PRS for ASD adjusted by sex and the first 4 principal components with school and individual nested as random effects. Blue shading indicates 95% CIs. Individuals with higher polygenic risk for ASD represented in the figure as +1SD (dark blue line) show a faster improvement on verbal WM over time compared to individuals with lower polygenic loads (-1SD, dashed light blue line).

### **Table S6**. Interaction effects between polygenic risk scores (PRS) for attention-deficit hyperactivity disorder (ADHD) and autism spectrum disorder (ASD) and sex on cognitive 1-year trajectories adjusting by age and the first four genetic principal components.

| **Outcome** | **PRS** | | **N** | **P-value interaction** |
| --- | --- | --- | --- | --- |
| **Verbal WM (*d'*)** | **ADHD** | *P_T_ < 0.01* | 4684 | 0.475 |
|  |  | *P_T_ < 0.05* | 4684 | 0.344 |
|  |  | *P_T_ < 0.1* | 4684 | 0.405 |
|  |  | *P_T_ < 0.5* | 4684 | 0.430 |
|  |  | *P_T_ < 1* | 4684 | 0.517 |
|  | **ASD** | *P_T_ < 0.01* | 4684 | 0.718 |
|  |  | *P_T_ < 0.05* | 4684 | 0.870 |
|  |  | *P_T_ < 0.1* | 4684 | 0.598 |
|  |  | *P_T_ < 0.5* | 4684 | 0.904 |
|  |  | *P_T_ < 1* | 4684 | 0.991 |
| **Numerical WM (*d'*)** | **ADHD** | *P_T_ < 0.01* | 4668 | 0.320 |
|  |  | *P_T_ < 0.05* | 4668 | 0.094 |
|  |  | *P_T_ < 0.1* | 4668 | 0.222 |
|  |  | *P_T_ < 0.5* | 4668 | 0.522 |
|  |  | *P_T_ < 1* | 4668 | 0.465 |
|  | **ASD** | *P_T_ < 0.01* | 4668 | 0.123 |
|  |  | *P_T_ < 0.05* | 4668 | 0.391 |
|  |  | *P_T_ < 0.1* | 4668 | 0.377 |
|  |  | *P_T_ < 0.5* | 4668 | 0.556 |
|  |  | *P_T_ < 1* | 4668 | 0.516 |
| **HRTSE** | **ADHD** | *P_T_ < 0.01* | 4646 | 0.211 |
|  |  | *P_T_ < 0.05* | 4646 | 0.918 |
|  |  | *P_T_ < 0.1* | 4646 | 0.479 |
|  |  | *P_T_ < 0.5* | 4646 | 0.597 |
|  |  | *P_T_ < 1* | 4646 | 0.583 |
|  | **ASD** | *P_T_ < 0.01* | 4646 | 0.948 |
|  |  | *P_T_ < 0.05* | 4646 | 0.183 |
|  |  | *P_T_ < 0.1* | 4646 | 0.681 |
|  |  | *P_T_ < 0.5* | 4646 | 0.372 |
|  |  | *P_T_ < 1* | 4646 | 0.492 |

### **Table S7**. Sensitivity analysis testing the association results between polygenic risk scores (PRS) for attention-deficit hyperactivity disorder (ADHD) and autism spectrum disorder (ASD) and cognitive outcomes including HRTSE (N=1,487), verbal (N=1,330) and numerical WM performance (N= 1,316), excluding 174 children diagnosed with ADHD. All analyses were adjusted by age, sex and the first four genetic principal components.

| **Outcome** | **PRS** | | **N** | **Beta coefficient (95% CIs)** | **P-uncorrec.** | **P-FDR** | **ΔR^2^** |
| --- | --- | --- | --- | --- | --- | --- | --- |
| **Verbal WM (*d'*)** | **ADHD** | *P_T_ < 0.01* | 1372 | -0.079 (-0.132; -0.027) | 0.003 | 0.028 | 0.057 |
|  |  | *P_T_ < 0.05* | 1372 | -0.055 (-0.108; -0.002) | 0.044 | 0.148 | 0.054 |
|  |  | *P_T_ < 0.1* | 1372 | -0.077 (-0.131; -0.024) | 0.005 | 0.028 | 0.057 |
|  |  | *P_T_ < 0.5* | 1372 | -0.085 (-0.138; -0.032) | 0.002 | 0.026 | 0.058 |
|  |  | *P_T_ < 1* | 1372 | -0.086 (-0.139; -0.033) | 0.002 | 0.026 | 0.058 |
|  | **ASD** | *P_T_ < 0.01* | 1372 | -0.013 (-0.067; 0.042) | 0.648 | 0.879 | 0.051 |
|  |  | *P_T_ < 0.05* | 1372 | 0.015 (-0.038; 0.069) | 0.577 | 0.879 | 0.051 |
|  |  | *P_T_ < 0.1* | 1372 | 0.023 (-0.03; 0.076) | 0.404 | 0.879 | 0.052 |
|  |  | *P_T_ < 0.5* | 1372 | 0.012 (-0.041; 0.065) | 0.658 | 0.879 | 0.051 |
|  |  | *P_T_ < 1* | 1372 | 0.011 (-0.043; 0.064) | 0.698 | 0.879 | 0.051 |
| **Numerical WM (*d'*)** | **ADHD** | *P_T_ < 0.01* | 1361 | -0.035 (-0.088; 0.017) | 0.189 | 0.554 | 0.018 |
|  |  | *P_T_ < 0.05* | 1361 | -0.078 (-0.131; -0.025) | 0.004 | 0.028 | 0.023 |
|  |  | *P_T_ < 0.1* | 1361 | -0.065 (-0.118; -0.012) | 0.017 | 0.072 | 0.021 |
|  |  | *P_T_ < 0.5* | 1361 | -0.062 (-0.115; -0.009) | 0.022 | 0.082 | 0.021 |
|  |  | *P_T_ < 1* | 1361 | -0.065 (-0.118; -0.012) | 0.017 | 0.072 | 0.021 |
|  | **ASD** | *P_T_ < 0.01* | 1361 | 0.009 (-0.044; 0.063) | 0.733 | 0.879 | 0.017 |
|  |  | *P_T_ < 0.05* | 1361 | 0.001 (-0.052; 0.054) | 0.965 | 0.965 | 0.017 |
|  |  | *P_T_ < 0.1* | 1361 | 0.021 (-0.032; 0.073) | 0.443 | 0.879 | 0.017 |
|  |  | *P_T_ < 0.5* | 1361 | 0.016 (-0.037; 0.068) | 0.562 | 0.879 | 0.017 |
|  |  | *P_T_ < 1* | 1361 | 0.021 (-0.032; 0.074) | 0.435 | 0.879 | 0.017 |
| **HRTSE** | **ADHD** | *P_T_ < 0.01* | 1364 | 0.733 (-3.517; 4.982) | 0.736 | 0.879 | 0.146 |
|  |  | *P_T_ < 0.05* | 1364 | 0.288 (-3.993; 4.57) | 0.895 | 0.947 | 0.146 |
|  |  | *P_T_ < 0.1* | 1364 | -0.668 (-4.979; 3.643) | 0.762 | 0.879 | 0.146 |
|  |  | *P_T_ < 0.5* | 1364 | 0.316 (-3.96; 4.592) | 0.885 | 0.947 | 0.146 |
|  |  | *P_T_ < 1* | 1364 | 0.232 (-4.059; 4.523) | 0.916 | 0.947 | 0.146 |
|  | **ASD** | *P_T_ < 0.01* | 1364 | -2.045 (-6.376; 2.287) | 0.356 | 0.879 | 0.147 |
|  |  | *P_T_ < 0.05* | 1364 | -2.773 (-7.034; 1.488) | 0.203 | 0.554 | 0.147 |
|  |  | *P_T_ < 0.1* | 1364 | -0.746 (-4.979; 3.487) | 0.73 | 0.879 | 0.146 |
|  |  | *P_T_ < 0.5* | 1364 | -1.261 (-5.493; 2.972) | 0.56 | 0.879 | 0.146 |
|  |  | *P_T_ < 1* | 1364 | -1.439 (-5.686; 2.808) | 0.508 | 0.879 | 0.147 |
| NOTE: *P_T_*, significance threshold for inclusion of variants in the polygenic score; CI; Confidence Interval; P-uncorr., uncorrected P-value; P-FDR, false discovery rate adjusted P-value; difference between the R² of the full model (PRS + covariates) compared to the R² of the model including only covariates. | | | | | | | |

# References

Bellgrove, M. A., Hester, R., & Garavan, H. (2004). The functional neuroanatomical correlates of response variability: Evidence from a response inhibition task. *Neuropsychologia*, *42*(14), 1910–1916. https://doi.org/10.1016/j.neuropsychologia.2004.05.007

Forns, J., Esnaola, M., López, M., Suades, E., Alvarez, M., Julvez, J., … Sunyer, J. (2014). The n-back Test and the Attentional Network Task as measures of child neuropsychological development in epidemiological studies. *Neuropsychology*, *28*(4), 519–529. https://doi.org/10.1037/neu0000085

Haatveit, B. C., Sundet, K., Hugdahl, K., Ueland, T., Melle, I., & Andreassen, O. A. (2010). The validity of d prime as a working memory index: results from the “Bergen n-back task.” *J Clin Exp Neuropsychol*, *32*(8), 871–880. https://doi.org/921109856 [pii]10.1080/13803391003596421

MacDonald, S. W. S., Nyberg, L., & Bäckman, L. (2006). Intra-individual variability in behavior: links to brain structure, neurotransmission and neuronal activity. *Trends in Neurosciences*, *29*(8), 474–480. https://doi.org/10.1016/j.tins.2006.06.011

Rueda, M. R., Fan, J., McCandliss, B. D., Halparin, J. D., Gruber, D. B., Lercari, L. P., & Posner, M. I. (2004). Development of attentional networks in childhood. *Neuropsychologia*, *42*(8), 1029–1040. https://doi.org/10.1016/j.neuropsychologia.2003.12.012

Shelton, J. T., Elliott, E. M., & Matthews, R. A. (2011). The Relationships of Working Memory, Secondary Memory, and General Fluid Intelligence: Working Memory is Special Jill, *36*(3), 813–820. https://doi.org/10.1037/a0019046.The

Wickens, T. D. (2002). *Elementary signal detection theory* . Oxford, New York: Oxford UP.
